# The Crucial Triad: Endothelial Glycocalyx, Oxidative Stress, and Inflammation in Cardiac Surgery—Exploring the Molecular Connections

**DOI:** 10.3390/ijms252010891

**Published:** 2024-10-10

**Authors:** Božena Ćurko-Cofek, Matej Jenko, Gordana Taleska Stupica, Lara Batičić, Antea Krsek, Tanja Batinac, Aleksandra Ljubačev, Marko Zdravković, Danijel Knežević, Maja Šoštarič, Vlatka Sotošek

**Affiliations:** 1 Department of Physiology, Immunology and Pathophysiology, Faculty of Medicine, University of Rijeka, Braće Branchetta 20, 51000 Rijeka, Croatia; 2 Clinical Department of Anaesthesiology and Surgical Intensive Care, University Medical Centre, Zaloska 7, 1000 Ljubljana, Slovenia; matej.jenko@kclj.si (M.J.); taleskagordana@gmail.com (G.T.S.); maja.sostaric@kclj.si (M.Š.); 3 Medical Faculty, University of Ljubljana, Vrazov Trg 2, 1000 Ljubljana, Slovenia; 4 Department of Medical Chemistry, Biochemistry and Clinical Chemistry, Faculty of Medicine, University of Rijeka, Braće Branchetta 20, 51000 Rijeka, Croatia; lara.baticic@uniri.hr; 5 Faculty of Medicine, University of Rijeka, Braće Branchetta 20, 51000 Rijeka, Croatia; antea.krsek@student.uniri.hr; 6 Department of Clinical Medical Sciences I, Faculty of Health Studies, University of Rijeka, Viktora Cara Emina 2, 51000 Rijeka, Croatia; tanjabatinac@net.hr (T.B.); vlatkast@uniri.hr (V.S.); 7 Department of Surgery, Faculty of Medicine, University of Rijeka, Braće Branchetta 20, 51000 Rijeka, Croatia; aleksandra.ljubacev@uniri.hr; 8 Department of Anaesthesiology, Intensive Care and Pain Management, University Medical Centre Maribor, Ljubljanska Ulica 5, 2000 Maribor, Slovenia; markozdravkovic@gmail.com; 9 Department of Anesthesiology, Reanimatology, Emergency and Intensive Care Medicine, University of Rijeka, Braće Branchetta 20, 51000 Rijeka, Croatia; danijel.knezevic2@uniri.hr

**Keywords:** cardiac surgery, endothelium, endothelial dysfunction, endothelial glycocalyx, inflammation, oxidative stress

## Abstract

Since its introduction, the number of heart surgeries has risen continuously. It is a high-risk procedure, usually involving cardiopulmonary bypass, which is associated with an inflammatory reaction that can lead to perioperative and postoperative organ dysfunction. The extent of complications following cardiac surgery has been the focus of interest for several years because of their impact on patient outcomes. Recently, numerous scientific efforts have been made to uncover the complex mechanisms of interaction between inflammation, oxidative stress, and endothelial dysfunction that occur after cardiac surgery. Numerous factors, such as surgical and anesthetic techniques, hypervolemia and hypovolemia, hypothermia, and various drugs used during cardiac surgery trigger the development of systemic inflammatory response and the release of oxidative species. They affect the endothelium, especially endothelial glycocalyx (EG), a thin surface endothelial layer responsible for vascular hemostasis, its permeability and the interaction between leukocytes and endothelium. This review highlights the current knowledge of the molecular mechanisms involved in endothelial dysfunction, particularly in the degradation of EG. In addition, the major inflammatory events and oxidative stress responses that occur in cardiac surgery, their interaction with EG, and the clinical implications of these events have been summarized and discussed in detail. A better understanding of the complex molecular mechanisms underlying cardiac surgery, leading to endothelial dysfunction, is needed to improve patient management during and after surgery and to develop effective strategies to prevent adverse outcomes that complicate recovery.

## 1. Introduction

Cardiac surgery began to develop at the beginning of the 20th century with the discovery of heparin, and protamine, and the development of extracorporeal circulation. Most cardiac surgery would be impossible without cardiopulmonary bypass (CPB) which allows adequate perfusion of the end-organ during the arrested (and protected) heart. It also provides the surgeon a bloodless field to safely perform the procedure on the heart and/or great vessels. Venous blood is collected via a venous cannula (double-stage or bi-caval) in the venous reservoir of the CPB device. It passes through an oxygenator, a heat exchanger, and filter and is returned as arterialised blood via an aortic cannula, which is usually placed in the ascending aorta. A separate suction pump draws blood from the surgical field to the CPB device, and another pump delivers a cardioplegic solution to arrest the heart and protect the myocard.

Since the first successful use in 1953 by John H. Gibbon Jr. [1], the CPB components have been modified several times to achieve the most physiological configuration and minimize the complications [2].

Although rigid venous reservoirs allow for better management of venous air and easier handling during CPB compared to collapsible “baggy” reservoirs, their silicone anti-foam components have been found to increase activation of blood cells and the incidence of microembolism formation [3,4]. In addition, the originally used bubble oxygenators were replaced by membrane oxygenators. It has been shown that they enable better blood gas management, produce fewer microemboli, and have less reactivity with blood components [5,6]. Newer polymethylpentene oxygenators also reduce plasma leakage during prolonged CPB [7].

Heparin-coated circuits have been developed with the intention of reducing the dose of systemic heparin and its adverse effects on coagulation homeostasis and inflammation [8,9,10,11,12]. Phosphorylcholine coating and other surface-modifying additives have also been introduced with the idea of better biocompatibility and thus reduce the inflammatory response [13,14,15]. Perfusion temperature is also related to the release of inflammatory mediators, with the highest levels observed with normothermic CPB, while lower levels were observed with hypothermic CPB [16,17].

Ultrafiltration/hemofiltration is used to remove excess fluid and low molecular weight particles from the plasma during CPB. Reduction in complement activation, pro-inflammatory cytokines, and clinical benefit have been noted in the pediatric population [18,19,20] but has not shown improvement in clinical outcomes or reduction in inflammation, which has been observed in adult patients [21]. Cytokine hemoadsorption (CytosorbTM) reduces the concentration of pro-inflammatory cytokines with improved postoperative hemodynamic and metabolic functions. The clinical benefit is manifested in the less frequent occurrence of acute respiratory distress syndrome and shorter postoperative ventilation times [22,23,24]. However, the issue of CPB-related tissue/organ dysfunction and inflammation associated with CPB still exists today. The term “whole-body inflammatory response” has been introduced to describe CPB-related inflammatory responses [25]. Thus, surgical trauma, contact of blood with non-endothelial surfaces, ischemia–reperfusion injury, and endotoxemia contribute to systemic inflammatory response syndrome (SIRS). It is a condition that occurs with varying intensity after cardiac surgery with CPB and influences the development of postoperative complications [26].

The standard mode of CPB is non-pulsatile flow, which is the most commonly used. While pulsatile CPB flow is possible, and logically, one would expect it to be a less detrimental to end-organ function, as it mimics the innate pulsatility of cardiovascular system, the results published today are conflicting [27,28,29,30]. Currently, the use of pulsatile CPB is recommended in the 2019 EACTS/EACTA/EBCP guidelines on CPB in adult cardiac surgery [31] in patients at a high risk of renal and pulmonary complications. Pahwa et al. [32] analyzed postoperative complications in 26.221 patients after cardiac surgery. Blood transfusions occurred in 47.3%, atrial fibrillation in 32%, prolonged ventilation in 8.9%, renal failure in 3.3%, reoperation for bleeding in 3.3%, and insertion of pacemaker/ICD in 3%. The study showed that pneumonia, renal failure, and stroke were associated with poor outcomes.

Recently, much attention has been paid to the effects of cardiac surgery on the vascular endothelium and the endothelial glycocalyx (EG) that cover it. Detachment of the EG during cardiac surgery is primarily triggered by ischemia–reperfusion injury, inflammatory responses, and oxidative stress. Degradation of EG during the perioperative period in cardiac surgery is also a well-known phenomenon, and given the role of endothelial function, its protection could improve patients’ outcomes [33].

In our previous work, much attention was paid to EG shedding in cardiac surgery, strategies to prevent this shedding [34], and the role of pre-existing cardiovascular disease that may affect the vascular endothelium and influence the outcome of cardiac surgery, as well as potential improvements in patient management before and during surgical procedures to minimize adverse events [35].

In this review, the complex mechanisms of endothelial dysfunction, oxidative stress, and inflammation and their interdependence in patients undergoing cardiac surgery were presented. The aim was to highlight the role of cardiac surgery as a trigger for each element of the crucial triad—EG damage, oxidative stress, and inflammation. We also highlight various factors that affect the endothelium and possible strategies to prevent its damage during surgery, which could lead to better patient outcomes.

## 2. EG Structure

EG is a gel-like structure on the luminal surface of the vascular endothelial cells that senses and transduces mechanical forces generated by blood flow [36]. In addition, it is involved as an electrical and mechanical barrier, vascular permeability, leukocyte–endothelial cell interaction, and vascular hemostasis [37,38]. Although EG covers the luminal surface of all blood vessels, its thickness and structure vary and depend mainly on the shear stress on the endothelial surface [39]. Therefore, we consider the EG as a dynamic structure, as it maintains a balance between the synthesis and shedding of its components [40].

The main components of the EG are glycoproteins, proteoglycans, and glycosaminoglycans (GAG) [41]. They are synthesized by the vascular endothelial cells, along with the numerous signalling molecules [42], and anchor EG to the endothelial cells, providing a matrix for other EG components, such as plasma proteins, cofactors, and enzymes such as albumin, thrombomodulin, superoxide dismutase, and xanthine-oxidoreductase, which contribute to EG homeostasis [42,43]. The glycoprotein orosomucoid is also incorporated into the EG matrix. Orosomucoid apparently interacts with the pores of the endothelial cells and thus reduce the amount of water that is filtered from plasma into the surrounding tissues [44]. Hidden in the matrix are also the adhesion molecules such as intercellular adhesion molecule 1 (ICAM-1), vascular cell adhesion molecule 1 (VCAM-1), and platelet endothelial cell adhesion molecule (PECAM-1), which are expressed on the surface of the endothelial cells. Thus, the matrix controls the aggregation of platelets and leukocytes, as well as leukodiapedesis, and prevents the initial steps in inflammation and coagulation in the blood vessel [35].

Glycoproteins are complex glycosylated molecules in which the carbohydrate groups are covalently bound to the protein. These carbohydrate side chains are short and capped with sialic acid, which contributes significantly to the negative surface charge of EG [45]. Proteoglycans consist of a core protein and at least one GAG chain attached to it [46]. The most important proteoglycan core proteins belong to the syndecan (syndecan-1 to syndecan-4) and glypican (glypican-1 to glypican-6) families, while the major GAGs are heparan sulphate, chondroitin sulphate, and hyaluronic acid [40]. GAGs have unbranched chains consisting of disaccharide units [47]. Heparan sulphate and chondroitin sulphate can have 50 to 150 repeating disaccharide units, while hyaluronic acid has 2000 to 25,000 units [40]. Hyaluronic acid is not bound to the core proteins but forms electrostatic bonds with other protein molecules of the EG, such as CD44, and thus provides structural support or may be present in soluble form [48].

Heparan sulphate makes up 50–90% of all GAGs in EG [43,48]. It shows structural differences, depending on cell type, tissue, and some pathological conditions [49]. The high proportion of heparan sulphate is consistent with its importance for the function of the EG. It is involved in the regulation of transmural fluid transport, as it helps to form the tight structure of the EG. Thus, macromolecules greater than 70 kDa cannot cross the EG barrier.

Heparan sulphate has been shown to be the primary sensor for the direction of shear stress [50] and a transducer of shear stress from the circulation to the endothelial intracellular space [51]. Shear stress activates endothelial nitric oxide synthase (eNOS) at the endothelial surface [50]. Studies have showed that enzymatic removal of heparan sulphate does not result in shear stress induced nitric oxide (NO) production [52].

The diverse function of heparan sulphate result from its ability to bind and modulate the activity of a variety of proteins, including growth factors, cytokines, morphogens, matrix structural proteins, enzymes or enzyme inhibitors, and surface proteins of the pathogens [52]. Thus, heparan sulphate, that is released into circulation by degraded EG, binds and inhibits the signalling pathways of inflammatory mediators during sepsis and have an anti-inflammatory effect. On the other hand, heparan sulphate fragments have been found to stimulate the release of pro-inflammatory cytokines and hyaluronan, which increases inflammation by binding to TLR-4 receptors [53].

**Figure 1:**
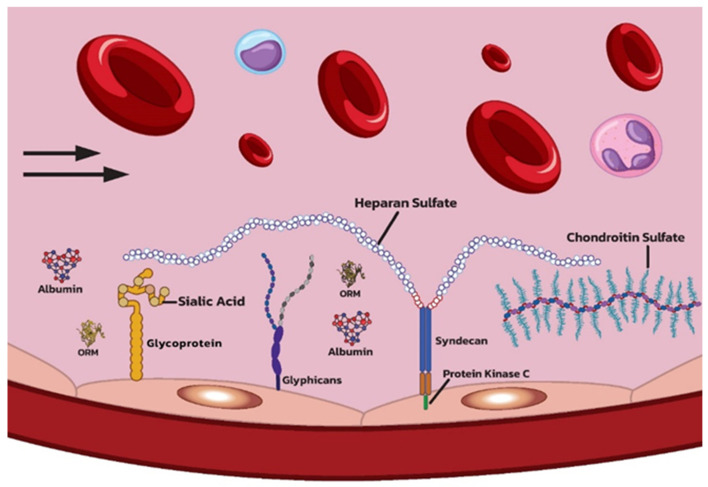
Schematic representation of the endothelial glycocalyx (EG) structure under physiological conditions. The EG covers the luminal surface of blood vessels. Some elements (glycoprotein, syndecan, and glypican) are bound to endothelial cells, while others (like heparan sulphate and chondroitin sulphate) have an indirect connection. Some molecules (like orosomucoid and albumins) are “trapped” within the matrix molecules. (ORM—orosomucoid).

Syndecan consists of a variable extracellular domain, a transmembrane domain, and a cytoplasmic tail [42]. The cytoplasmic tail is in contact with protein kinase C. Therefore, it can transmit information about the mechanical forces acting on the apical side of the vascular endothelial cell and initiate the appropriate intracellular signalling pathway [54] (Figure 1).

Syndecans interact with the cytoskeleton, thus acting as a principal effector in cell adhesion and cell shape change. Syndecan-1 has a tyrosine residue and thus interacts with cytoskeletal proteins, while syndecan-4, in combination with integrins, rearranges the actin stress fibres and adapts the cell for adhesion [55].

Glypicans are associated with the surface of the cell membrane, usually in lipid raft regions with many signalling molecules. Thus, glypican-1 acts as a coreceptor in signalling pathways and modulates them [56]. As mentioned above, the effect of the blood shear force leads to activation of eNOS, NO production, and vasodilation [34]. Glypican molecules mediate flow-induced eNOS activation. Recent studies have shown that inhibition of glypican-1 enhances the expression of inflammatory gene and monocyte adhesion but also inhibits NO expression, leading to impaired function of endothelial cells and inflammation [57].

The normal structure of EG is of the most importance when knowing its physiological functions within the vascular barrier. In case of ischemia or inflammation, the components of EG start to disintegrate and shed, leading to impaired microcirculation, fluid extravasation and edema, leukocyte and platelet adhesion, hypercoagulability, and loss of flow-dependent vasodilatation [58]. As a result of shedding, EG components enter the circulation, thus representing an effective tool for monitoring and evaluating EG function. It has been noted that syndecan subtypes 1 and 3 increase in critical conditions, such as sepsis. Hyaluronic acid and heparan sulphate are used as biomarkers of endothelial injury [59]. Shedding can be initiated by the influence of reactive oxygen species (ROS), inflammatory mediators and catecholamines, enzymes released by damaged tissue (heparanases, matrix metalloproteinases), ischemia, and acute hyperglycemia [60]. All these mechanisms can be induced during cardiac surgery with CPB.

Recent studies have shown that prolonged cardiopulmonary bypass can be associated with EG degradation [61,62] and suggested that in off-pump coronary artery bypass grafting (CABG) surgery time reduction, reduction in compression and movement of the heart during operation could decrease the degradation of EG [62]. Cardiac ischemia–reperfusion that occurs during open heart surgery [63] could lead to the degradation and shedding of EG [64,65,66,67,68] due to increased production of ROS and reactive nitrogen species (RNS) or inflammation [69,70,71]. The concentration of the soluble glycocalyx component syndecan-1 has been shown to increase in the systemic circulation even before aortic cross-clamping and the onset of ischemia [67,72]. In the case of ischemia followed by reperfusion under CPB, increased concentration of syndecan-1 and heparan sulphate can be found in the circulation [73]. The nucleosides adenosine and inosine have been proposed as mediators of ischemia-induced EC degradation. They are produced by the degradation of ATP and ADP during an insufficient oxygen supply [74]. Adenosine and inosine bind to adenosine receptors on the surface of mast cells in the human myocardium, which contain the enzyme heparanase stored in granules. When released, heparanase cleaves heparan sulphate from EG [73]. Therefore, stabilization of mast cells and blockade of adenosine receptors can be an option to inhibit post-ischemic shedding.

After injury, the EG can be repaired. It has been shown in animal models that the glycocalyx requires five to seven days to fully recover [33]. Clinical studies have shown that regeneration process can be even faster [56,75], suggesting that the regeneration and protection of EG are especially significant in the early perioperative period.

## 3. Molecular Mechanisms of EG Degradation in Cardiac Surgery

Vascular endothelial function can be damaged and impaired during cardiac surgery by several non-specific and specific factors that activate the inflammatory response. Non-specific factors include mechanical damage due to surgical techniques, turbulent blood flow, hypervolemia and hypovolemia due to transfusion or blood loss, and hypothermia [76,77]. The inflammatory response can be activated during CPB by direct contact with foreign surfaces of the CPB circuit, ischemia–reperfusion injury to different organs [76] such as the brain, heart, lungs, kidney, liver [78], and endotoxemia [79]. It has been shown that systemic endotoxin following CPB correlates with the degree of cardiovascular dysfunction [67,80]. It has been suggested that splanchnic hypoperfusion, common during and after CPB [81], may damage the mucosal barrier, leading to translocation of endotoxin [82]. Also, endotoxin may contaminate fluids that are routinely used during CPB, such as the cardioplegia and circuit priming fluid [83].

The main molecular mechanisms involved in detachment of EG, or glycocalyx shedding, include activation of proteases, which degrade glycocalyx components. At the same time, this degradation is further enhanced by increased concentrations of ROS and inflammatory cytokines.

### 3.1. Shear Stress

Through mechanosensation and mechanotransduction, the EG senses the shear stress of the blood flow and converts it into intracellular signals. Under normal physiological conditions, shear stress from laminar blood flow maintains the integrity of EG by regulating the synthesis of its components. However, the alteration of blood flow during cardiac surgery contributes to the degradation of the glycocalyx since disruption of blood flow and shear stress patterns can downregulate the expression of syndecan-1 and other EG components [55]. The shear-stress-induced signalling pathway is activated by endothelial cell junction proteins, such as PECAM-1. It is a cell-adhesion molecule and mechanosensor of endothelial cells that acts in a complex of different junctional proteins, including vascular endothelial cadherin (VE-cadherin) and vascular endothelial growth factor receptor 2 (VEGFR2) [35]. Heparan sulphate is crucial for endothelial mechanotransduction initiation in the early phase. PECAM-1 and the G protein Gαq/11 form a mechanosensitive complex containing endogenous heparan sulphate proteoglycans with a chondroitin sulphate chain which is central to the assembly of the complex and regulation of the flow response.

This mechanical disruption weakens the structure of the EG and makes it more susceptible to enzymatic and oxidative damage. Shear-induced NO production is a hallmark of endothelial mechanotransduction, which is central to flow-mediated vasodilatation [84]. The proteoglycan core protein glypican-1 transmits the fluid shear force sensed by GAG side chains to the cell surface mainly via heparan sulphate but not via chondroitin sulphate and syndecan-1, resulting in phosphorylation of eNOS to NO. Shear stress, which acts directly on glypican-1 can also trigger NO production in in vitro models [85]. Glypican-1 is the primary upstream sensor for shear stress, highlighting the role of PECAM-1 as a downstream mediator of shear-stress-induced NO formation. Treatment with heparinase blocked both the early and late phases of NO production, partially by disrupting heparan sulphate in complex with PECAM-1 [86].

Glycocalyx degradation is also mediated by exocytosis of Weibel–Palade bodies and secretory lysosomes, which are visible as patch loss or craters in the glycocalyx [87]. Weibel–Palade bodies store adhesion receptors for platelets (von Willebrand factor) and leukocytes (P-selectin). Therefore, the exocytosis Weibel–Palade body promotes platelet clumping and adhesion of leukocytes to vascular endothelial cells [88].

### 3.2. Protease Activation

**Figure 2:**
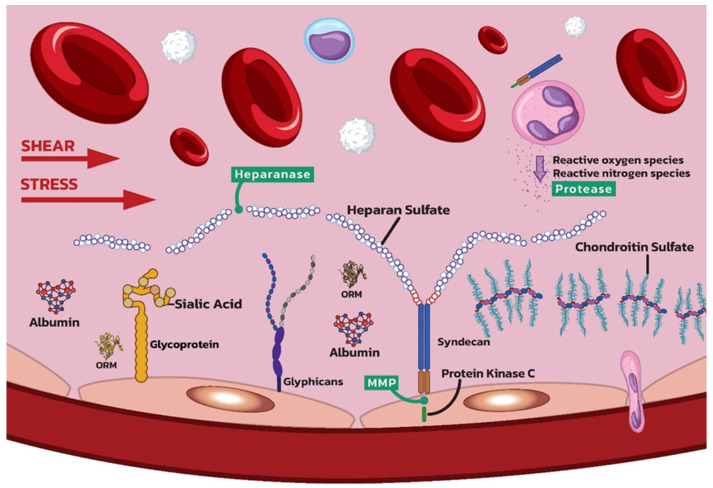
Schematic representation of EG exposed to shear stress. Note the detachment of heparan and chondroitin sulphate. Various proteases (primary matrix metalloproteinases (MMP), heparanase, and hyaluronidase) are released by activated leukocytes or induced by mechanical stress. Proteases cleave the core proteins of proteoglycans and glycoproteins in the endothelial glycocalyx, leading to their degradation. (ORM—orosomucoid).

One of the key mechanisms for glycocalyx detachment is the activation of various proteases/sheddases, including matrix metalloproteinases (MMPs), heparanase, and hyaluronidase. Other potential molecules with a similar detachment function are neutrophil elastase, thrombin, plasmin, tryptase, and cathepsin B [89]. They are frequently activated by ROS and pro-inflammatory cytokines [90] (Figure 2).

MMPs are a family of enzymes that degrade extracellular matrix components [91]. During cardiac surgery, ischemia–reperfusion injury leads to the activation of MMPs, particularly MMP-2 and MMP-9 [92,93], which are released by phagocytes [94]. These enzymes cleave the core proteins of proteoglycans and glycoproteins in the glycocalyx, leading to their degradation [95]. It is hypothesized that the MMPs cleave the syndecan ectodomain. Elevated MMPs levels have been associated with increased degradation of EG and the resulting vascular permeability and inflammation [96]. Doxycycline, a non-selective inhibitor of MMP activity, reduces glycocalyx detachment [94].

Heparanase specifically cleaves heparan sulphate, a major component of the EG. Ischemia–reperfusion injury and inflammation caused by cardiac surgery significantly increase heparanase activity [34]. The upregulation of heparanase not only degrades heparan sulphate but also promotes the release of growth factors and cytokines stored in the EG, thus exacerbating inflammatory responses and endothelial dysfunction [97].

Hyaluronidase degrades hyaluronic acid, another important component of the EG. Pro-inflammatory cytokines, such as tumour necrosis factor-alpha (TNF-α) and interleukin (IL)-1β, which increase during cardiac surgery, stimulate hyaluronidase activity. This degradation reduces the protective barrier function of the glycocalyx and increases endothelial permeability and leukocyte adhesion [98].

Vascular leakage and EG damage are significant concerns in CPB surgery. Therefore, several therapeutic interventions have been proposed to mitigate these issues. Imatinib, a tyrosine kinase inhibitor, has shown promising results in reducing vascular leakage and maintaining microcirculatory perfusion, potentially protecting the EG during CPB. Thus, it could improve outcomes, such as lowering markers of renal injury [99]. Another strategy to attenuate these effects in CPB is to target angiopoietin-2 (Ang-2), which is known for its role in endothelial hyperpermeability and capillary leak [100,101]. Although general strategies to treat vascular leakage in sepsis may be applicable, particularly to influence inflammatory pathways and endothelial barrier function [102], treatment of systemic capillary leak syndrome (SCLS) during CPB focuses on stabilizing the endothelial barrier and reducing capillary leakage [103]. Therapeutic agents such as corticosteroids, albumin, sphingosine-1-phosphate receptor agonists, and vasopressin have also been identified as potential treatments due to their role in reducing inflammation, restoring colloid osmotic pressure, and improving the integrity of the endothelial junction. Additional agents such as angiopoietin-1 (Ang-1), anti-inflammatory drugs, and antioxidants such as ascorbic acid offer additional therapeutic options. Furthermore, the protective effects of statins and the potential use of heparin and heparanase inhibitors to prevent glycocalyx degradation in CPB settings should be further investigated [102].

### 3.3. Oxidative Stress

Oxidative stress is the result of an imbalance between the production of ROS and their degradation by various antioxidants, leading to an excess of ROS or RNS that is associated with numerous pathophysiological processes [104,105]. Reactive species are normally produced in the body and act in balance with antioxidants, mainly as signalling molecules, that significantly influence cell growth, cell differentiation, and cell ageing [106]. However, when reactive species accumulate, they can cause cellular and molecular damage. Accumulation can be caused by external or internal causes. External causes include radiation, heavy metals, and long-lasting stress. The predominant internal production of reactive species is associated with mitochondria, cytochrome p450, and NADPH oxidases [106].

Oxidative stress is major contributor to the detachment of the EG during cardiac surgery. Some components of EG, such as GAGs, heparan sulphate and hyaluronic acid, are more susceptible to oxidative damage. Activated neutrophils produce ROS and RNS and release granules containing proteases responsible for degradation [107]. One of the most important sources is neutrophil-derived myeloperoxidase, which is bound to the negatively charged GAG side chains. The reperfusion process after ischemia generates ROS, including superoxide anions, hydrogen peroxide, and hydroxyl radicals. These ROS directly damage the EG by oxidizing its components, leading to structural degradation [108]. In addition, ROS activate redox-sensitive transcription factors such as the nuclear factor kappa-light-chain-enhancer of activated B cells (NF-κB), which upregulate the expression of pro-inflammatory cytokines and adhesion molecules, and further promote EG degradation [109].

Patients who have undergone cardiac surgery, either with or without CPB, are at risk of the production of ROS and RNS [110]. The species can cause further damage, both intraoperatively and postoperatively, leading to atrial fibrillation and ischemia, enhancing the need for fluid resuscitation, which in turn can overload the heart, induce N-terminal proBrain Natriuremic Peptide (NT-proBNP) production, and damage the EG. Therefore, perioperative oxidative stress should be considered in cardiac surgery as well as possible methods to reduce perioperative ROS production and the use of potential antioxidant therapies to limit the impact in this vulnerable patients [111].

Resveratrol is a potential antioxidant. There are no human clinical studies in cardiac surgery but with potential results in animal and human in vitro model studies [112,113]. Vitamin C is an antioxidant that is being researched in various areas, including cardiac surgery. In cardiac surgery, vitamin C effects are focused in the intraoperative and postoperative period including direct effects on the heart and other organs (lungs, kidneys), blood coagulation, and immune function [114,115,116], although some controversy with publication as bias has been noted [117]. Coenzyme Q10 is another potential treatment method that needs further studies and quality results [118]. Acetaminophen, as a more common treatment option, both for postoperative pain and its anti-inflammatory and antioxidant effects, has also been researched and showed positive results [119]. Various other dietary supplements have been researched but showed variable results [120,121,122].

Most of the results are not yet conclusive or only offer short-term benefits and require further research to confirm the results and reevaluate the protocols, doses used or long-term outcomes.

### 3.4. Ischemia–Reperfusion Injury

Ischemia–Reperfusion injury disrupts the EG [123], leading to tissue damage [48]. Cardiac ischemia–reperfusion injury can occur during percutaneous coronary angioplasty, CABG, and heart transplantation [63]. Studies have shown that cardiac surgery and CPB can lead to degradation of the EG and shedding of its components, such as syndecan-1 and heparan sulphate, into the bloodstream [65,80,124,125,126]. In cardiac surgery patients, early release of syndecan-1 and heparan sulphate was observed during reperfusion [72]. An inflammatory response due to CPB could result in EG component shedding even after off-pump CABG surgery [127] due to ischemia–reperfusion injury from temporary ligation of coronary arteries, reversible low cardiac output during surgery, or hypotension [69]. Ischemia–reperfusion injury to the brain, heart, lung, kidney, and liver [78] due to the aortic cross-clamping and restoration of perfusion after its release is associated with activation of an inflammatory response [77]. According to the evidence from animal studies of ischemia–reperfusion injury, the reduced thickness of EG can be observed as early as 5 min after reperfusion, and glycocalyx shedding leads to NO-mediated endothelium-dependent vasodilation [77,128].

As part of the crucial triad molecular circuit, ROS are important effectors in EG damage during ischemia–reperfusion injury [39]. It has been shown that administration of the antioxidative agent superoxide dismutase protects small vessels from ischemia–reperfusion damage and protects EG [129]. Elevated blood levels of EG components, such as syndecan-1 [130] and heparan sulphate [131], have been found in patients after CPB, survivors of cardiac arrest and acute coronary syndrome, suggesting that syndecan-1 as a core protein and heparan sulphate as a GAG side chain are affected in ischemia–reperfusion injury. Heparan sulphate shedding during ischemia–reperfusion injury is associated with increased vessel permeability, complement activation, thrombosis, and leukocyte infiltration into the damaged tissue [39]. Endothelial dysfunction, complement activation, and interaction of vascular endothelial cells with immune cells, such as neutrophils, occur as the earliest inflammatory response during ischemia–reperfusion injury [132]. Animal studies have shown that ischemia–reperfusion injury can result in EG shedding due to increased production of ROS and RNS or a secondary inflammatory response [71], leading to increased levels of syndecan-1 and heparan sulphate in the circulation [77,129].

Animal studies have also detected complement system involvement in ischemia–reperfusion injury [133]. Deposits of complement components C3b and C5b-9 have been detected in reperfused hearts of myocardial infarction patients [134], in association with the increase in syndecan-1 shedding [135]. It has been suggested that increased complement deposition and tissue injury might be caused by the loss of interaction of damaged EG with complement regulatory proteins in plasma, such as C1-inhibitor [136]. It has also been suggested that complement activation in ischemia–reperfusion injury may be due to the expression of neoantigens on the endothelial cell surface [137], which bind to naturally occurring IgM antibodies, leading to complement activation and tissue injury [138].

In addition to interacting with plasma proteins, EG plays a protective role in shielding cell surface adhesion molecules and limiting their interaction with immune cells [139]. It has been shown that damage to EG during myocardial infarction contributes to neutrophil and platelet adhesion to vessel walls and vascular edema [140].

Damage to EG during the neutrophil-mediated immune response may occur by enzymatic degradation by MMPs and hyaluronidase or by oxidative stress [39]. In addition, elastase, cathepsins, and MMPs released by neutrophils can cleave endothelial cell–cell junctions, resulting in damaged junctional integrity and vascular leakage [141].

Activated neutrophils can form web-like structures of decondensed chromatin, histones, and cytoplasmic and granular proteins known as neutrophil extracellular traps (NETs), which have been shown to be released in peripheral vascular disease, myocardial infarction and stroke [142]. Histones released from NET formation are highly cytotoxic to endothelial cells and, in reaction with EG, cause barrier dysfunction and microvascular leakage [143]. Histone levels have been shown to correlate with infarct size [144].

Both neutrophil activation and complement deposition are tightly linked and play a central role in EG damage during ischemia–reperfusion injury [39]. Animal studies on complement receptor 5a knock-out mouse model of myocardial infarction showed a reduction in neutrophil migration to postischemic myocardium and diminished expression of MMP-9 [145].

The destruction and shedding of EG during ischemia–reperfusion injury is an important process involving the metalloproteinase family, especially MMPs, but also many different sheddases [39,146,147]. Sheddases and sulfatases remove entire GAG side chains and proteoglycans or alter the sulfation pattern of GAG side chains, thereby altering the EG [39]. MMPs, which have been shown to cleave whole proteoglycans, are also released during ischemia–reperfusion injury, possibly by cardiomyocytes and neutrophils [148,149]. Shedding of syndecan-1 during ischemia–reperfusion injury is a consequence of MMPs upregulation and downregulation of the MMPs tissue inhibitors [150].

Studies have linked MMP-3 and MMP-9 to cardiovascular disease, showing their elevated levels in patients with ischemic heart disease and atherosclerotic plaques [39,151,152]. The degradation and shedding of EG are facilitated by ischemia–reperfusion injury in patients after cardiac surgery and CPB, resulting in the heparan sulphate and syndecan-1 release into the circulation [127]. Therefore, elevated levels of glycocalyx components (syndecan-1, heparan sulphate, hyaluronan) can be detected in the blood and urine of patients following the activation of sheddases, heparinase, MMPs, and hyaluronidase, which are probably of endothelial origin [76]. Cleavage of hyaluronan has been observed in ischemic stroke patients, suggesting the involvement of sheddase hyaluronidase in EG degradation during ischemia–reperfusion injury [153]. In addition, it has been shown that atrial natriuretic peptide [154], tryptase-β [155], and heparinase [156] result in soluble syndecan-1 increase in ischemia–reperfusion injury [157].

### 3.5. Inflammation

**Figure 3:**
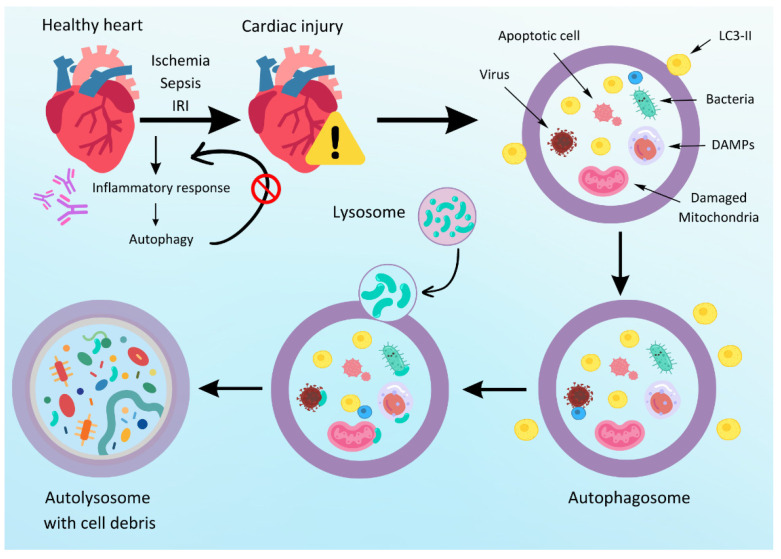
Inflammatory response in heart damage. This Figure represents the autophagy process in response to cardiac injury, such as ischemia, sepsis, or ischemia–reperfusion injury. Following cardiac injury, an inflammatory response is triggered, which activates the autophagy pathway. Damaged cells, including apoptotic cells, viruses, bacteria, damage-associated molecular patterns (DAMPs), and damaged mitochondria, are encapsulated in a double-membrane structure called an autophagosome. LC3-II is a marker protein involved in the formation of the autophagosome. The autophagosome then fuses with a lysosome, forming an autolysosome. The lysosomal enzymes degrade the encapsulated cell debris within the autolysosome, leading to its breakdown and recycling, thereby aiding cellular recovery and homeostasis.

Inflammatory processes that occur during cardiac surgery represent a complex physiological response that can be triggered by numerous factors. They are orchestrated by a variety of different dynamics, linked by a number of molecular mechanisms that include tissue injury, CPB-induced stress, ischemia–reperfusion injury, and systemic immune activation. These multifaceted processes are mediated by the release of damage-associated molecular patterns (DAMPs), activation of pattern recognition receptors (PRRs), generation of ROS, cytokine production, immune cell recruitment, and complement activation (Figure 3) with a growing body of scientific evidence emphasizing new insights in this field [158,159].

Scientific and clinical research on inflammation-reducing techniques during cardiac surgery, such as hemoadsorption and temperature management, shows promising results in improving patient outcomes by mitigating the inflammatory response [22,113,160,161].

#### 3.5.1. Tissue Injury

In the first line, the tissue injury caused by the surgical trauma leads to cell damage and the release of intracellular contents, which further activates the immune system [162]. The surgical procedure disrupts the skin, resulting in damage to the tissue and organ barriers. This leads to the activation of inflammatory mediators. Even in minimally invasive surgeries that attempt to minimize tissue trauma, it is essentially impossible to completely prevent the release of these mediators [163].

According to McCully and Moser, a ray of chemokines is harbouring in human skin [164]. In a mouse model with surgical incision, C-X-C motif ligand 1 (CXCL1), also known as keratinocyte chemoattractant (KC), is the first key chemokine released within the first 12 h, followed by macrophage inflammatory protein-2 (MIP-2) which is released after 24 h. Both chemokines attract neutrophils [165]. Although the differences between rodents and humans are well known, surgical manipulation also causes the release of both anti-inflammatory cytokines and pro-inflammatory cytokines in humans [166]. According to the murine research, macrophages are usually present in skin tissue, and the influx of neutrophils occurs within 24 h of an incision before declining sharply within eight days. Before the influx of neutrophils into murine aseptic wounds, endothelial barrier permeability increases. Even after neutrophil removal, permeability remains high, indicating that it is not controlled by the cells alone during the inflammatory responses. Therefore, the cellular inflammatory response seems to contribute to increased endothelial barrier permeability but cannot cause it alone [167]. Furthermore, not only neutrophils but also platelets are involved in the regulation of endothelial permeability, as shown by the reduced thrombin-mediated endothelial permeability after platelet depletion in the skin incision model [168].

#### 3.5.2. CPB-Induced Stress

Secondly, CPB is a critical component of many cardiac surgeries, as blood comes into contact with the artificial surfaces of the CPB circuit, causing additional stress and inflammatory reactions. Direct contact of blood with CPB leads to activation of the complement cascade, mainly via an alternative pathway. In addition, the complement is activated during tissue reperfusion and heparin neutralization with protamine [169].

The heparin–protamine complex can trigger the inflammatory response not only by complement activation but also via histamine release, thromboxane and NO production, and antibody formation [170,171]. The heparin–protamine complex activates complement mainly through the classical (c4a) pathway [169]. Delayed activation of complement could be observed in the first five days after the cardiac surgery, and it is associated with C reactive protein due to heparin–protamine complexes [172].

The importance of complement activation during the inflammatory response after cardiac surgery is highlighted by the action of complement-specific inhibitors which prevent platelet, neutrophils, and/or monocyte activation [173]. In addition, the mechanical forces in the CPB circuit can cause hemolysis and activate immune cells [174]. The activated immune system further releases DAMPs, subsequently triggering PRRs [166]. The human vascular endothelium expresses PRRs with variable distribution through main arteries, and their increased expression in endothelial dysfunction due to diabetes, arterial hypertension, hypercholesterolemia, and hyperuricemia allows for endothelial activation by PRR ligands [175].

The temporary restriction of the blood supply during surgery leads to tissue hypoxia and cellular stress. The endothelial cells lining the blood vessels are activated and express adhesion molecules that recruit further immune cells and contribute to vascular permeability [174]. Activated lymphocytes secrete pro-inflammatory cytokines. They play a central role in the inflammatory process following cardiac surgery, which is characterized by an early increase in TNF-α and IL-1β and a later increase in IL-6 and IL-8. Pro-inflammatory mediators initiate various signal transduction pathways and activate the transcription factor NF-κB, leading to gene transcription and translation of proteins required for endothelial cell activation, such as adhesion molecules (e.g., E-selectin, intercellular adhesion molecule-1) and cytokines (e.g., IL-8) [77].

It has been suggested that the clinical prognosis after CPB depends on the fine balance between the production of pro-inflammatory and anti-inflammatory cytokines [26]. It has been shown that an increase in pro-inflammatory cytokines correlates with a poorer outcome after cardiac surgery. Mortality rate after pediatric cardiac surgery has been shown to correlate with serum IL-6 levels [176]. A significant increase in cytokine concentration has been found in cardiac surgery patients who develop SIRS. A significant increase in IL-8 and IL-18 was seen in non-survivors compared to survivors [77].

There are various efforts to reduce CPB-induced inflammation. Some of these methods include lowering cytokine levels through hemoadsorption techniques and manipulating temperature control during surgery. These treatments are designed to reduce the immune response and improve patient outcomes after cardiac surgery [177].

#### 3.5.3. Temperature Management and Reperfusion

Similarly, temperature management and restoration of blood flow in previously ischemic tissues contribute to inflammation [178]. Hypothermia is commonly used during CPB to reduce metabolic demands and protect organs from ischemic damage. Nevertheless, a more balanced approach is needed to avoid adverse consequences, considering recent work that has drawn attention to the intricacy of temperature control and underscored hazards tied to both cooling-down and warming-up processes [26]. During CPB, hypothermia can effectively reduce the overall inflammatory response and ROS formation. The presence of such a protective mechanism is affected by reducing cellular metabolic rate because of lowering body temperature. Similarly minimized during such processes, however, are treatments towards ischemia–reperfusion injury. These processes are associated with decreased activation of pro-inflammatory signalling pathways and decreased cellular endothelial activity, which limits the migration of leukocytes into the tissue by decreasing vascular permeability [179,180].

Rewarming, an essential step post-CPB, must be carefully controlled to prevent a sudden increase in inflammatory mediators. Rapid rewarming, on the other hand, can lead to another phenomenon known as “rewarming shock”, which means a transient increase in ROS production and a pronounced inflammatory response. During this period, increased endothelial dysfunction, augmented vascular permeability, and increased potential of invasion by immune cells, such as neutrophils or macrophages, can be triggered, leading to further tissue damage [181].

To date, temperature management strategies have been developed to achieve optimal results by precisely controlling the cooling and reheating rates to achieve thermal stability with minimal inflammatory reactions. For instance, gradual rewarming reduces the occurrence of rewarming shock and reduces some adverse effects associated with rapid temperature changes [77]. In addition, pharmacological methods have been used in an attempt to modulate the inflammatory cascade during the rewarming process. In this context, studies on ROS scavengers, pro-inflammatory cytokine inhibitors, and endothelial function enhancers are underway for their potential to reduce inflammation and improve recovery after surgery [182,183].

#### 3.5.4. SIRS

SIRS is defined as an extreme inflammatory reaction of the entire body, usually caused by a severe infection or trauma. In cardiac surgery, this is of particular interest, as the procedures themselves are associated with severe stress and tissue damage. An early systemic reaction can lead to complications during postoperative recovery and ultimately increase morbidity and mortality [26]. At the molecular level, SIRS is recognized during CPB, where pro-inflammatory cytokines such as IL-6, TNF-α, and IL-1β are released, amplifying the inflammatory response [137]. SIRS can lead to extensive inflammation affecting multiple organs, potentially resulting in organ dysfunction. Likewise, CPB is known to activate the complement system and thus contributes to the inflammatory processes [183].

Preventive measures for SIRS in cardiac surgery include several strategies aimed at minimizing the inflammatory response triggered by surgical procedures. One of these is the optimization of surgical techniques to reduce tissue trauma. In addition, less invasive techniques can significantly reduce the inflammatory burden on the body. The use of biocompatible materials in CPB circuits is important, as they reduce the contact activation of blood components involved in triggering the inflammatory response during CPB [184].

The inflammatory response is also modulated by pharmacological agents, such as anti-inflammatory drugs like corticosteroids or other immunomodulatory drugs. New pharmacological interventions are currently being tested to further minimize the risk and severity of SIRS in cardiac surgery patients [185].

To prevent such a serious condition as SIRS as a consequence of cardiac surgery, early treatment and various approaches to prevent EG damage and shedding during cardiac surgery, especially in the early postoperative period, are the subject of intensive research [186,187,188,189,190]. There is increasing evidence that maintenance of fluid balance and administration of glycocalyx-sparing “restrictive” fluid regimens [191], volatile anesthesia, maintenance of normal plasma and albumin concentrations [192], and normoglycemia [193] can minimize glycocalyx damage. Fresh frozen plasma [194] and the administration of human albumin [195] have been shown to have a strong protective and regenerative effect on the EG.

While sevoflurane can preserve EG in ischemia–reperfusion injury, a high dose of propofol can lead to EG injury [188,196]. Since hyperglycemia can lead to endothelial dysfunction and cause glycocalyx shedding [40], metformin has been suggested to enhance glycocalyx density and thickness, thus improving glycocalyx function. Furthermore, insulin and metformin have been shown to increase NO synthesis and arterial dilatation [197], while empagliflozin preserves glycocalyx integrity [198].

## 4. Conclusions

The triad of EG dysfunction, oxidative stress, and inflammation in patients undergoing cardiac surgery shares common pathways and is linked at the molecular level. In this complex interplay, we have identified some key molecules, but their interdependence and trends need to be better explored in the hope of identifying potential clinical interventions that would reduce EG shedding, oxidative stress, and/or inflammation. Understanding these mechanisms and their consequences during cardiac surgery is critical for developing strategies to mitigate their effects and improve patient outcomes. Some of the potential interventions to prevent the activation of the crucial triad include improvements in surgical techniques to minimize surgical trauma, improvements in CPB device, and anesthesia protocols, including optimal regulation of patient hemodynamic stability, maintenance of normothermia, and use of medications during cardiac surgery and in the postoperative period.

## References

[1] Gibbon J.H. (1954). Application of a mechanical heart and lung apparatus to cardiac surgery. Minn. Med..

[2] Holman W.L., Timpa J., Kirklin J.K. (2022). Origins and Evolution of Extracorporeal Circulation: JACC Historical Breakthroughs in Perspective. J. Am. Coll. Cardiol..

[3] Orenstein J.M., Sato N., Aaron B., Buchholz B., Bloom S. (1982). Microemboli observed in deaths following cardiopulmonary bypass surgery: Silicone antifoam agents and polyvinyl chloride tubing as source of emboli. Hum. Pathol..

[4] Schonberger J.P.A.M., Everts P.A.M., Hoffman J.J. (1995). Systemic blood activation with open and closed venous reservoirs. Ann. Thorac. Surg..

[5] Blauth C.I., Smith P.L., Arnold J.V., Jagoe J.R., Wootton R., Taylor K.M., Loop F.D. (1990). Influence of oxygenator type on the prevalence and extent of micro-emboli retinal ischemia during cardio-pulmonary bypass: Assessment by digital image analysis. J. Thorac. Cardiovasc. Surg..

[6] Pearson D.T. (1990). Gas exchange; bubble and membrane oxygenators. Semin. Thorac. Cardiovasc. Surg..

[7] Wiesenack C., Wiesner G., Keyl C., Gruber M., Philipp A., Ritzka M., Prasser C., Taeger K. (2002). In vivo uptake and elimination of isoflurane by different membrane oxygenators during cardiopulmonary bypass. Anesthesiology.

[8] Videm V., Mollnes T.E., Fosse E., Mohr B., Bergh K., Hagve T.A., Aasen A.O., Svennevig J.L. (1999). Heparin-coated cardiopulmonary bypass equipment, I: Biocompatibility markers and development of complications in a high-risk population. J. Thorac. Cardiovasc. Surg..

[9] Goudeau J.-J., Clermont G., Guillery O., Lemaire-Ewing S., Musat A., Vernet M., Vergely C., Guiguet M., Rochette L., Girard C. (2007). In high-risk patients, combination of antiinflammatory procedures during cardiopulmonary bypass can reduce incidences of inflammation and oxidative stress. J. Cardiovasc. Pharmacol..

[10] Plötz F.B., van Oeveren W., Hultquist K.A., Miller C., Bartlett R.H., Wildevuur C.R. (1992). A heparin-coated circuit reduces complement activation and the release of leukocyte inflammatory mediators during extracorporeal circulation in a rabbit. Artif. Organs..

[11] Mangoush O., Purkayastha S., Hajyahia S., Kinross J., Hayward M., Bartolozzi F., Darzi A., Athanasiou T. (2007). Heparin-bonded circuits versus nonheparin-bonded circuits: An evaluation of their effect on clinical outcomes. Eur. J. Cardiothorac. Surg..

[12] Svenmarker S., Häggmark S., Jansson E., Lindholm R., Appelblad M., Sandström E., Åberg T. (2002). Use of heparin-bonded circuits in cardiopulmonary bypass improves clinical outcome. Scand. Cardiovasc. J..

[13] Thiara A.S., Andersen V.Y., Videm V., Mollnes T.E., Svennevig K., Hoel T.N., Fiane A. (2010). Comparable biocompatibility of phisio- and bioline-coated cardiopulmonary bypass circuits indicated by the inflammatory response. Perfusion.

[14] Sohn N., Marcoux J., Mycyk T., Krahn J., Meng Q. (2009). The impact of different biocompatible coated cardiopulmonary bypass circuits on inflammatory response and oxidative stress. Perfusion.

[15] Dekker N.A.M., Veerhoek D., van Leeuwen A.L.I., Vonk A.B.A., van den Brom C.E., Boer C. (2020). Microvascular alterations during cardiac surgery using a heparin or phosphorylcholine-coated circuit. J. Cardiothorac. Vasc. Anesth..

[16] Menasché P.H. (2001). The systemic factor: The comparative roles of cardiopulmonary bypass and off-pump surgery in the genesis of patient injury during and following cardiac surgery. Ann. Thorac. Surg..

[17] Menasché P., Peynet J., Heffner-Cavaillon N., Carreno M.-P., de Chaumaray T., Dillisse V., Faris B., Piwnica A., Bloch G., Tedgui A. (1995). Influence of temperature on neutrophil trafficking during clinical cardiopulmonary bypass. Circulation.

[18] Naik S.K., Knight A., Elliot M. (1991). A prospective randomized study of a modified technique of ultrafiltration during pediatric open-heart surgery. Circulation.

[19] Journois D., Pouard P., Greeley W.J., Mauriat P., Vouhe P., Safran D. (1994). Hemofiltration in pediatric cardiac surgery. Anesthesiology.

[20] Watanabe T., Sakai Y., Mayumi T., Shimomura T., Song M.H., Tajima K., Suenaga Y., Kawaradani Y., Saito Y., Yamada T. (1998). Effect of ultrafiltration during cardiopulmonary bypass for pediatric cardiac surgery. Artif. Organs..

[21] Grunefelder J., Zund G., Schoeberlein A., Maly F.E., Schurr U., Guntli S., Fischer K., Turina M. (2000). Modified ultrafiltration lowers adhesion molecules and cytokine levels after cardiopulmonary bypass without clinical relevance in adults. Eur. J. Cardiothorac. Surg..

[22] Bernardi M.H., Rinoesl H., Dragosits K., Ristl R., Hoffelner F., Opfermann P., Lamm C., Preißing F., Wiedemann D., Hiesmayr M.J. (2016). Effect of hemoadsorption during cardiopulmonary bypass surgery—A blinded, randomized, controlled pilot study using a novel adsorbent. Crit. Care..

[23] Gleason T.G., Argenziano M., Bavaria J.E., Kane L.C., Coselli J.S., Engelman R.M., Tanaka K.A., Awad A., Sekela M.E., Zwischenberger J.B. (2019). Hemoadsorption to reduce plasma-free hemoglobin during cardiac surgery: Results of REFRESH I pilot study. Semin. Thorac. Cardiovasc. Surg..

[24] Doukas P., Hellfritsch G., Wendt D., Magliani M., Barbati M.E., Jalaie H., Jacobs M.J., Gombert A. (2023). Intraoperative hemoadsorption (cytosorb TM) during open thoracoabdominal aortic repair: A pilot randomized controlled trial. J. Clin. Med..

[25] Kouchoukos N.T., Blackstone E.H., Hanley F.L., Kirklin J.K. (2012). Chapter 2: Hypothermia, circulatory arrest, and cardiopulmonary bypass. Kirklin/Barratt-Boyes Cardiac Surgery E-Book.

[26] Paparella D., Yau T.M., Young E. (2002). Cardiopulmonary bypass induced inflammation: Pathophysiology and treatment. An update. Eur. J. Cardiothorac. Surg..

[27] Tan A., Newey C., Falter F. (2022). Pulsatile Perfusion during Cardiopulmonary Bypass: A Literature Review. J. Extra Corpor. Technol..

[28] Sievert A., Sistino J. (2012). A meta-analysis of renal benefits to pulsatile perfusion in cardiac surgery. J. Extra. Corpor. Technol..

[29] Nam M.J., Lim C.H., Kim H.-J., Kim Y.H., Choi H., Son H.S., Lim H.J., Sun K. (2015). A meta-analysis of renal function after adult cardiac surgery with pulsatile perfusion. Artif. Organs..

[30] Lim C.H., Nam M.J., Lee J.S., Kim H.J., Kim J.Y., Shin H.W., Lee H.W., Sun K. (2015). A meta-analysis of pulmonary function with pulsatile perfusion in cardiac surgery. Artif. Organs..

[31] Kunst G., Milojevic M., Boer C., De Somer F.M.J.J., Gudbjartsson T., van der Goor J., Jones T.J., Lomivorotov V., Merkle F., Ranucci M. (2019). 2019 EACTS/EACTA/EBCP guidelines on cardiopulmonary bypass in adult cardiac surgery. Br. J. Anaesth..

[32] Pahwa S., Bernabei A., Schaff H., Stulak J., Greason K., Pochettino A., Daly R., Dearani J., Bagameri G., King K. (2021). Impact of postoperative complications after cardiac surgery on long-term survival. J. Card. Surg..

[33] Potter D.R., Jiang J., Damiano E.R. (2009). The recovery time course of the endothelial cell glycocalyx in vivo and its implications in vitro. Circ. Res..

[34] Knežević D., Ćurko-Cofek B., Batinac T., Laškarin G., Rakić M., Šoštarič M., Zdravković M., Šustić A., Sotošek V., Batičić L. (2023). Endothelial Dysfunction in Patients Undergoing Cardiac Surgery: A Narrative Review and Clinical Implications. J. Cardiovasc. Dev. Dis..

[35] Kršek A., Batičić L., Ćurko-Cofek B., Batinac T., Laškarin G., Miletić-Gršković S., Sotošek V. (2024). Insights into the Molecular Mechanism of Endothelial Glycocalyx Dysfunction during Heart Surgery. Curr. Issues Mol. Biol..

[36] Weinbaum S., Cancel L.M., Fu B.M., Tarbell J.M. (2021). The Glycocalyx and Its Role in Vascular Physiology and Vascular Related Diseases. Cardiovasc. Eng. Technol..

[37] Tay E.A., Vijayakumar V., Morales R.F., Lee E.S., Teo A. (2024). Protecting the endothelial glycocalyx in COVID-19. PLoS Pathog..

[38] Qu R., Du W., Li S., Li W., Wei G., Chen Z., Gao H., Shi S., Zou L., Li H. (2024). Destruction of vascular endothelial glycocalyx during formation of pre-metastatic niches. Heliyon.

[39] Milusev A., Rieben R., Sorvillo N. (2022). The Endothelial Glycocalyx: A Possible Therapeutic Target in Cardiovascular Disorders. Front. Cardiovasc. Med..

[40] Kaur G., Harris N.R. (2023). Endothelial glycocalyx in retina, hyperglycemia, and diabetic retinopathy. Am. J. Physiol. Cell Physiol..

[41] Hahn R.G., Patel V., Dull R.O. (2021). Human glycocalyx shedding: Systematic review and critical appraisal. Acta Anaesthesiol. Scand..

[42] Foote C.A., Soares R.N., Ramirez-Perez F.I., Ghiarone T., Aroor A., Manrique-Acevedo C., Padilla J., Martinez-Lemus L. (2022). Endothelial Glycocalyx. Compr. Physiol..

[43] Zhao F., Zhong L., Luo Y. (2021). Endothelial glycocalyx as an important factor in composition of blood-brain barrier. CNS Neurosci. Ther..

[44] Luo Z., Lei H., Sun Y., Liu X., Su D.-F. (2015). Orosomucoid, an acute response protein with multiple modulating activities. J. Physiol. Biochem..

[45] Kincses A., Santa-Maria A.R., Walter F.R., Dér L., Horányi N., Lipka D.V., Valkai S., Deli M.A., Der A. (2020). A chip device to determine surface charge properties of confluent cell monolayers by measuring streaming potential. Lab. Chip..

[46] Ferreira G., Taylor A., Mensah S.A. (2024). Deciphering the triad of endothelial glycocalyx, von Willebrand Factor, and P-selectin in inflammation-induced coagulation. Front. Cell Dev. Biol..

[47] Cosgun Z.C., Fels B., Kusche-Vihrog K. (2020). Nanomechanics of the endothelial glycocalyx: From structure to function. Am. J. Pathol..

[48] Pillinger N.L., Kam P.C.A. (2017). Endothelial glycocalyx: Basic science and clinical implications. Anaesth. Intensive Care..

[49] Annaval T., Wild R., Cretinon Y., Sadir R., Vives R.R., Lortat-Jacob H. (2020). Heparan Sulfate Proteoglycans Biosynthesis and Post Synthesis Mechanisms Combine Few Enzymes and Few Core Proteins to Generate Extensive Structural and Functional Diversity. Molecules.

[50] Hu Z., Cano I., D’Amore P.A. (2021). Update on the Role of the Endothelial Glycocalyx in Angiogenesis and Vascular Inflammation. Front. Cell Dev. Biol..

[51] Pretorius D., Richter R.P., Anand T., Cardenas J.C., Richter J.R. (2022). Alterations in heparan sulfate proteoglycan synthesis and sulfation and the impact on vascular endothelial function. Matrix Biol. Plus..

[52] Ricard-Blum S., Vivès R.R., Schaefer L., Götte M., Merline R., Passi A., Heldin P., Magalhães A., Reis C.A., Skandalis S.S. (2024). A biological guide to glycosaminoglycans: Current perspectives and pending questions. FEBS J..

[53] Oshima K., Haeger S.M., Hippensteel J.A., Herson P.S., Schmidt E.P. (2018). More than a biomarker: The systemic consequences of heparan sulfate fragments released during endothelial surface layer degradation. Pulm. Circ..

[54] Gopal S. (2020). Syndecans in Inflammation at a Glance. Front. Immunol..

[55] Villalba N., Baby S., Yuan S.Y. (2021). The Endothelial Glycocalyx as a Double-Edged Sword in Microvascular Homeostasis and Pathogenesis. Front. Cell. Dev. Biol..

[56] Pan J., Ho M. (2021). Role of glypican-1 in regulating multiple cellular signaling pathways. Am. J. Physiol. Cell Physiol..

[57] Mahmoud M., Mayer M., Cancel L.M., Bartosch A.M., Mathews R., Tarbell J.M. (2021). The glycocalyx core protein Glypican 1 protects vessel wall endothelial cells from stiffness-mediated dysfunction and disease. Cardiovasc. Res..

[58] Belousoviene E., Kiudulaite I., Pilvinis V., Pranskunas A. (2021). Links between endothelial glycocalyx changes and microcirculatory parameters in septic patients. Life.

[59] Iba T., Levy J.H. (2019). Derangement of the endothelial glycocalyx in sepsis. J. Thromb. Haemost..

[60] Barry M., Pati S. (2022). Targeting repair of the vascular endothelium and glycocalyx after traumatic injury with plasma and platelet resuscitation. Matrix Biol. Plus.

[61] Robich M., Ryzhov S., Kacer D., Palmeri M., Peterson S.M., Quinn R.D., Carter D., Sheppard F., Hayes T., Sawyer D.B. (2020). Prolonged Cardiopulmonary Bypass is Associated with Endothelial Glycocalyx Degradation. J. Surg. Res..

[62] Wang J., Wu Y. (2024). Mass intraoperative endothelial glycocalyx shedding affects postoperative systemic inflammation response. BMC Anesthesiol..

[63] Reffelmann T., Kloner R.A. (2006). The no-reflow phenomenon: A basic mechanism of myocardial ischemia and reperfusion. Basic. Res. Cardiol..

[64] Rehm M., Bruegger D., Christ F., Conzen P., Thiel M., Jacob M., Chappell D., Stoeckelhuber M., Welsch U., Reichart B. (2007). Shedding of the endothelial glycocalyx in patients undergoing major vascular surgery with global and regional ischemia. Circulation.

[65] Koning N.J., Vonk A.B.A., Vink H., Boer C. (2016). Side-by-Side Alterations in Glycocalyx Thickness and Perfused Microvascular Density During Acute Microcirculatory Alterations in Cardiac Surgery. Microcirculation.

[66] Wu Q., Gao W., Zhou J., He G., Ye J., Fang F., Luo J., Wang M., Xu H., Wang W. (2019). Correlation between acute degradation of the endothelial glycocalyx and microcirculation dysfunction during cardiopulmonary bypass in cardiac surgery. Microvasc. Res..

[67] Bruegger D., Rehm M., Abicht J., Paul J.O., Stoeckelhuber M., Pfirrmann M., Reichart B., Becker B.F., Christ F. (2009). Shedding of the endothelial glycocalyx during cardiac surgery: On-pump versus off-pump coronary artery bypass graft surgery. J. Thorac. Cardiovasc. Surg..

[68] Svennevig K., Hoel T., Thiara A., Kolset S., Castelheim A., Mollnes T., Brosstad F., Fosse E., Svennevig J. (2008). Syndecan-1 plasma levels during coronary artery bypass surgery with and without cardiopulmonary bypass. Perfusion.

[69] Chappell D., Bruegger D., Potzel J., Jacob M., Brettner F., Vogeser M., Conzen P., Becker B.F., Rehm M. (2014). Hypervolemia increases release of atrial natriuretic peptide and shedding of the endothelial glycocalyx. Crit. Care.

[70] Mulivor A.W., Lipowsky H.H. (2004). Inflammation- and ischemia-induced shedding of venular glycocalyx. Am. J. Physiol. Heart Circ. Physiol..

[71] Warren O.J., Smith A.J., Alexiou C., Rogers P.L., Jawad N., Vincent C., Darzi A.W., Athanasiou T. (2009). The inflammatory response to cardiopulmonary bypass: Part 1—Mechanisms of pathogenesis. J. Cardiothorac. Vasc. Anesth..

[72] Passov A., Schramko A., Salminen U.S., Aittomäki J., Andersson S., Pesonen E. (2021). Endothelial glycocalyx during early reperfusion in patients undergoing cardiac surgery. PLoS ONE.

[73] Becker B.F., Jaco M., Leipert S., Salmon A.H.J., Chappell D. (2015). Degradation of the endothelial glycocalyx in clinical settings: Searching for the sheddases. Br. J. Clin. Pharmacol..

[74] Becker B.F., Fischer J., Hartmann H., Chen C.C., Sommerhoff C.P., Tschoep J., Conzen P.C., Annecke T. (2011). Inosine, not adenosine, initiates endothelial glycocalyx degradation in cardiac ischemia and hypoxia. Nucleos. Nucleot. Nucl..

[75] Goncharov N.V., Nadeev A.D., Jenkins R.O., Avdonin P.V. (2017). Markers and Biomarkers of Endothelium: When Something Is Rotten in the State. Oxid. Med. Cell Longev..

[76] Dogné S., Flamion B. (2020). Endothelial glycocalyx Impairment in Disease: Focus on Hyaluronan Shedding. Am. J. Pathol..

[77] Warltier D.C., Laffey J.G., Boylan J.F., Cheng D.C. (2002). The systemic inflammatory response to cardiac surgery: Implications for the anesthesiologist. Anesthesiology.

[78] Zhang M., Liu Q., Meng H., Duan H., Liu X., Wu J., Gao F., Wang S., Tan R., Yuan J. (2024). Ischemia-reperfusion injury: Molecular mechanisms and therapeutic targets. Signal Transduct. Target Ther..

[79] Tarbell J.M., Cancel L.M. (2016). The glycocalyx and its significance in human medicine. J. Intern. Med..

[80] Dekker N.A.M., Veerhoek D., Koning N.J., van Leeuwen A.L.I., Elbers P.W.G., van den Brom C.E., Vonk A.B.A., Boer C. (2019). Postoperative microcirculatory perfusion and endothelial glycocalyx shedding following cardiac surgery with cardiopulmonary bypass. Anaesthesia.

[81] Spiess B.D. (2017). Heparin: Effects upon the Glycocalyx and Endothelial Cells. J. Extra Corpor Technol..

[82] Koning N.J., Simon L.E., Asfar P., Baufreton C., Boer C. (2014). Systemic microvascular shunting through hyperdynamic capillaries after acute physiological disturbances following cardiopulmonary bypass. Am. J. Physiol. Heart Circ. Physiol..

[83] Cabrales P., Vázquez B.Y., Tsai A.G., Intaglietta M. (2007). Microvascular and capillary perfusion following glycocalyx degradation. J. Appl. Physiol..

[84] Pahakis M.Y., Kosky J.R., Dull R.O., Tarbell J.M. (2007). The role of endothelial glycocalyx components in mechanotransduction of fluid shear stress. Biochem. Biophys. Res. Commun..

[85] Zeng Y., Zhang X.F., Fu B.M., Tarbell J.M. (2018). The Role of Endothelial Surface Glycocalyx in Mechanosensing and Transduction. Adv. Exp. Med. Biol..

[86] Osawa M., Masuda M., Kusano K., Fujiwara K. (2002). Evidence for a role of platelet endothelial cell adhesion molecule-1 in endothelial cell mechanosignal transduction: Is it a mechanoresponsive molecule?. J. Cell Biol..

[87] Zullo J.A., Fan J., Azar T.T., Yen W., Zeng M., Chen J., Ratliff B.B., Song J., Tarbell J.M., Goligorsky M.S. (2016). Exocytosis of Endothelial Lysosome-Related Organelles Hair-Triggers a Patchy Loss of Glycocalyx at the Onset of Sepsis. Am. J. Pathol..

[88] Naß J., Terglane J., Gerke V. (2021). Weibel Palade Bodies: Unique Secretory Organelles of Endothelial Cells that Control Blood Vessel Homeostasis. Front. Cell Dev. Biol..

[89] Patterson E.K., Cepinskas G., Fraser D.D. (2022). Endothelial Glycocalyx Degradation in Critical Illness and Injury. Front. Med..

[90] Jin J., Fang F., Gao W., Chen H., Wen J., Wen X., Wen X., Chen J. (2021). The Structure and Function of the Glycocalyx and Its Connection With Blood-Brain Barrier. Front. Cell. Neurosci..

[91] Oberleithner H. (2014). Vascular endothelium: A vulnerable transit zone for merciless sodium. Nephrol. Dial. Transplant..

[92] Huang L., Tian W., Chen X., Xu H., Wanbing D., Zhang Y., Wu X., Yu W., Tian J., Su D. (2022). Peripheral Neutrophils-Derived Matrix Metallopeptidase-9 Induces Postoperative Cognitive Dysfunction in Aged Mice. Front. Aging Neurosci..

[93] Serraino G.F., Jiritano F., Costa D., Ielapi N., Battaglia D., Bracale U.M., Mastroroberto P., Andreucci M., Serra R. (2023). Metalloproteinases in Cardiac Surgery: A Systematic Review. Biomolecules.

[94] Lipowsky H.H., Lescanic A. (2013). The effect of doxycycline on shedding of the glycocalyx due to reactive oxygen species. Microvasc. Res..

[95] Turner N.A., Porter K.E. (2012). Regulation of myocardial matrix metalloproteinase expression and activity by cardiac fibroblasts. IUBMB Life.

[96] Sieve I., Münster-Kühnel A.K., Hilfiker-Kleiner D. (2018). Regulation and function of endothelial glycocalyx layer in vascular diseases. Vascul. Pharmacol..

[97] Langjahr P., Díaz-Jiménez D., De la Fuente M., Rubio E., Golenbock D., Bronfman F.C., Quera R., Gonzales M.-J., Hermoso M.A. (2014). Metalloproteinase-Dependent TLR2 Ectodomain Shedding is Involved in Soluble Toll-Like Receptor 2 (sTLR2) Production. PLoS ONE.

[98] Dogné S., Flamion B., Caron N. (2018). Endothelial Glycocalyx as a Shield Against Diabetic Vascular Complications: Involvement of Hyaluronan and Hyaluronidases. Arterioscler. Thromb. Vasc. Biol..

[99] Koning N.J., Vonk A.B., Boonstra P.W., van Barneveld L.J., van Leeuwen P.A., Kesecioglu J. (2018). Imatinib reduces vascular leakage and preserves microcirculatory perfusion during experimental cardiopulmonary bypass. J. Cardiovasc. Pharmacol..

[100] Wollborn J., Mayer K., Dürbeck M., Dinkla M., Ebmeyer S., Kopp R. (2023). Angiopoietin-2 as a prognostic marker and potential therapeutic target in cardiopulmonary bypass surgery. J. Thorac. Cardiovasc. Surg..

[101] Dekker N.A.M., Veerhoek D., Koning N.J., van Meurs M., Vink H., van Leeuwen P.A. (2019). Plasma angiopoietin-2 levels are associated with impaired endothelial glycocalyx and predict kidney injury after cardiac surgery with cardiopulmonary bypass. Sci. Rep..

[102] McMullan R.R., Parker S.J., Moore L.J. (2024). Management strategies for vascular leak in sepsis: Potential applications to cardiopulmonary bypass. Crit. Care Med..

[103] Aşgün H.F., Oğuz S. (2023). Systemic capillary leak syndrome in cardiac surgery: Diagnosis and management strategies. Turk. J. Med. Sci..

[104] D’Oria R., Schipani R., Leonardini A., Natalicchio A., Perrini S., Cignarelli A., Laviola L., Giorgino F. (2020). The Role of Oxidative Stress in Cardiac Disease: From Physiological Response to Injury Factor. Oxid. Med. Cell. Longev..

[105] Toro-Pérez J., Rodrigo R. (2021). Contribution of oxidative stress in the mechanisms of postoperative complications and multiple organ dysfunction syndrome. Redox. Rep..

[106] Aranda-Rivera A.K., Cruz-Gregorio A., Arancibia-Hernández Y.L., Hernández-Cruz E.Y., Pedraza-Chaverri J. (2022). RONS and Oxidative Stress: An Overview of Basic Concepts. Oxygen.

[107] Song J.W., Goligorsky M.S. (2018). Perioperative implication of the endothelial glycocalyx. Korean J. Anesthesiol..

[108] Higashi Y., Maruhashi T., Noma K., Kihara Y. (2014). Oxidative stress and endothelial dysfunction: Clinical evidence and therapeutic implications. Trends Cardiovasc. Med..

[109] Berdiaki A., Neagu M., Spyridaki I., Kuskov A., Perez S., Nikitovic D. (2023). Hyaluronan and Reactive Oxygen Species Signaling-Novel Cues from the Matrix?. Antioxidants.

[110] Karu I., Taal G., Zilmer K., Pruunsild C., Starkopf J., Zilmer M. (2010). Inflammatory/oxidative stress during the first week after different types of cardiac surgery. Scand. Cardiovasc. J..

[111] Zakkar M., Ascione R., James A.F., Angelini G.D., Suleiman M.S. (2015). Inflammation, oxidative stress and postoperative atrial fibrillation in cardiac surgery. Pharmacol. Ther..

[112] Usta E., Mustafi M., Walker T., Ziemer G. (2011). Resveratrol suppresses apoptosis in intact human cardiac tissue—In vitro model simulating extracorporeal circulation. J. Cardiovasc. Surg..

[113] Zhu Y., Feng B., He S., Su Z., Zheng G. (2018). Resveratrol combined with total flavones of hawthorn alleviate the endothelial cells injury after coronary bypass graft surgery. Phytomedicine.

[114] Hill A., Wendt S., Benstoem C., Neubauer C., Meybohm P., Langlois P., Adhikari N.K., Heyland D.K., Stoppe C. (2018). Vitamin C to Improve Organ Dysfunction in Cardiac Surgery Patients-Review and Pragmatic Approach. Nutrients.

[115] Hu X., Yuan L., Wang H., Li C., Cai J., Hu Y., Ma C. (2017). Efficacy and safety of vitamin C for atrial fibrillation after cardiac surgery: A meta-analysis with trial sequential analysis of randomized controlled trials. Int. J. Surg..

[116] Polymeropoulos E., Bagos P., Papadimitriou M., Rizos I., Patsouris E., Τoumpoulis I. (2016). Vitamin C for the Prevention of Postoperative Atrial Fibrillation after Cardiac Surgery: A Meta-Analysis. Adv. Pharm. Bull..

[117] Hemilä H. (2017). Publication bias in meta-analysis of ascorbic acid for postoperative atrial fibrillation. Am. J. Health. Syst. Pharm..

[118] de Frutos F., Gea A., Hernandez-Estefania R., Rabago G. (2015). Prophylactic treatment with coenzyme Q10 in patients undergoing cardiac surgery: Could an antioxidant reduce complications? A systematic review and meta-analysis. Interact. Cardiovasc. Thorac. Surg..

[119] Xiong C., Jia Y., Wu X., Zhao Y., Yuan S., Yan F., Sessler D.I. (2023). Early Postoperative Acetaminophen Administration and Severe Acute Kidney Injury After Cardiac Surgery. Am. J. Kidney Dis..

[120] Ali-Hassan-Sayegh S., Mirhosseini S.J., Tahernejad M., Mahdavi P., Shahidzadeh A., Karimi-Bondarabadi A.A., Dehghan A., Rahimizadeh E., Haddad F., Ghodratipour Z. (2016). Impact of antioxidant supplementations on cardio-renal protection in cardiac surgery: An updated and comprehensive meta-analysis and systematic review. Cardiovasc. Ther..

[121] Wendt S., Schomburg L., Manzanares W., Stoppe C. (2019). Selenium in Cardiac Surgery. Nutr. Clin. Pract..

[122] Geng J., Qian J., Si W., Cheng H., Ji F., Shen Z. (2017). The clinical benefits of perioperative antioxidant vitamin therapy in patients undergoing cardiac surgery: A meta-analysis. Interact. Cardiovasc. Thorac. Surg..

[123] Seal J.B., Gewertz B.L. (2005). Vascular dysfunction in ischemia-reperfusion injury. Ann. Vasc. Surg..

[124] Bol M.E., Huckriede J.B., van de Pas K.G.H., Delhaas T., Lorusso R., Nicolaes G.A.F., Sels J.E.M., van de Poll M.C.G. (2022). Multimodal measurement of glycocalyx endothelial glycocalyx radiation during coronary artery bypass grafting. Front. Med..

[125] Pesonen E., Passov A., Andersson S., Suojaranta R., Niemi T., Raivio P., Salmenperä M., Schramko A. (2019). Glycocalyx Degradation and Inflammation in Cardiac Surgery. J. Cardiothorac. Vasc. Anesth..

[126] Wu M.-Y., Yiang G.-T., Liao W.-T., Tsai A.P.-Y., Cheng Y.-L., Cheng P.-W., Li C.-Y., Li C.-J. (2018). Current Mechanistic Concepts in Ischemia and Reperfusion Injury. Cell. Physiol. Biochem..

[127] Squiccimarro E., Stasi A., Lorusso R., Paparella D. (2022). Narrative review of the systemic inflammatory reaction to cardiac surgery and cardiopulmonary bypass. Artif. Organs..

[128] Platts S.H., Linden J., Duling B.R. (2003). Rapid modification of the glycocalyx caused by ischemia-reperfusion is inhibited by adenosine A2A receptor activation. Am. J Physiol..

[129] Rubio-Gayosso I., Platts S.H., Duling B.R. (2006). Reactive oxygen species mediate modification of glycocalyx during ischemia-reperfusion injury. Am. J. Physiol..

[130] Miranda C.H., de Carvalho Borges M., Schmidt A., Marin-Neto J.A., Pazin-Filho A. (2016). Evaluation of the endothelial glycocalyx damage in patients with acute coronary syndrome. Atherosclerosis.

[131] Grundmann S., Fink K., Rabadzhieva L., Bourgeois N., Schwab T., Moser M., Bode C., Busch H.J. (2012). Perturbation of the endothelial glycocalyx in post cardiac arrest syndrome. Resuscitation.

[132] Morgan B.P. (2000). The complement system: An overview. Methods Mol. Biol..

[133] Diepenhorst G.M.P., van Gulik T.M., Hack C.E. (2009). Complement-mediated ischemia-reperfusion injury: Lessons learned from animal and clinical studies. Ann. Surg..

[134] Nijmeijer R., Krijnen P.A., Assink J., Klaarenbeek M.A., Lagrand W.K., Veerhuis R., Visser C.A., Meijer C.J., Niessen H.W., Hack C.E. (2004). C-reactive protein and complement depositions in human infarcted myocardium are more extensive in patients with reinfarction or upon treatment with reperfusion. Eur. J Clin. Investig..

[135] Chaban V., Nakstad E.R., Stær-Jensen H., Schjalm C., Seljeflot I., Vaage J., Lundqvist C., Benth J.Š., Sunde K., Mollnes T.E. (2021). Complement activation is associated with poor outcome after out-of-hospital cardiac arrest. Resuscitation.

[136] Langford-Smith A., Day A.J., Bishop P.N., Clark S.J. (2015). Complementing the sugar code: Role of GAGs and sialic acid in complement regulation. Front. Immunol..

[137] Eltzschig H.K., Eckle T. (2011). Ischemia and reperfusion—From mechanism to translation. Nat. Med..

[138] Busche M.N., Pavlov V., Takahashi K., Stahl G.L. (2009). Myocardial ischemia and reperfusion injury is dependent on both IgM and mannose-binding lectin. Am. J. Physiol..

[139] Reitsma S., Slaaf D.W., Vink H., van Zandvoort M.A., oude Egbrink M.G.A. (2007). The endothelial glycocalyx: Composition, functions, and visualization. Pflugers. Arch..

[140] Chappell D., Heindl B., Jacob M., Annecke T., Chen C., Rehm M., Conzen P., Becker B.F. (2011). Sevoflurane reduces leukocyte and platelet adhesion after ischemia reperfusion by protecting the endothelial glycocalyx. Anesthesiology.

[141] Ma Y., Yang X., Chatterjee V., Meegan J.E., Beard R.S., Yuan S.Y. (2019). Role of neutrophil extracellular traps and vesicles in regulating vascular endothelial permeability. Front. Immunol..

[142] Sorvillo N., Cherpokova D., Martinod K., Wagner D.D. (2019). Extracellular DNA NET-works with dire consequences for health. Circ. Res..

[143] Meegan J.E., Yang X., Beard R.S., Jannaway M., Chatterjee V., Taylor-Clark T.E., Yuan S.Y. (2018). Citrullinated histone 3 causes endothelial barrier dysfunction. Biochem. Biophys. Res. Commun..

[144] Shah M., He Z., Rauf A., Kalkhoran S.B., Heiestad C.M., Stensløkken K.-O., Parish C.R., Soehnlein O., Arjun S., Davidson S.M. (2021). Extracellular histones are a target in myocardial ischaemia–reperfusion injury. Cardiovasc. Res..

[145] Mueller M., Herzog C., Larmann J., Schmitz M., Hilfiker-Kleiner D., Gessner J.E., Theilmeier G. (2013). The receptor for activated complement factor 5 (C5aR) conveys myocardial ischemic damage by mediating neutrophil transmigration. Immunobiology.

[146] Yang X., Meegan J.E., Jannaway M., Coleman D.C., Yuan S.Y. (2018). A disintegrin and metalloproteinase 15-mediated glycocalyx shedding contributes to vascular leakage during inflammation. Cardiovasc. Res..

[147] Ramnath R.D., Butler M.J., Newman G., Desideri S., Russell A., Lay A.C., Neal C.R., Qiu Y., Fawaz S., Onions K.L. (2020). Blocking matrix metalloproteinase-mediated syndecan-4 shedding restores the endothelial glycocalyx and glomerular filtration barrier function in early diabetic kidney disease. Kidney Int..

[148] Romanic A.M., Harrison S.M., Bao W., Burns-Kurtis C.L., Pickering S., Gu J., Grau E., Mao J., Sathe G.M., Ohlstein E.H. (2002). Myocardial protection from ischemia/reperfusion injury by targeted deletion of matrix metalloproteinase-9. Cardiovasc. Res..

[149] Lalu M.M., Pasini E., Schulze C.J., Ferrari-Vivaldi M., Ferrari-Vivaldi G., Bachetti T., Schulz R. (2005). Ischaemia–reperfusion injury activates matrix metalloproteinases in the human heart. Eur. Heart J..

[150] Ali M.M., Mahmoud A.M., Le Master E., Levitan I., Phillips S.A. (2019). Role of matrix metalloproteinases and histone deacetylase in oxidative stress-induced degradation of the endothelial glycocalyx. Am. J. Physiol. Heart Circ. Physiol..

[151] Sun H., Zhang J., Zheng Y., Shang S. (2018). Expressions and clinical significance of factors related to acute coronary syndrome. J. Biol. Regul. Homeost. Agents..

[152] Reine T.M., Lanzalaco F., Kristiansen O., Enget A.R., Satchell S., Jenssen T.G., Kolset S.O. (2019). Matrix metalloproteinase-9 mediated shedding of syndecan-4 in glomerular endothelial cells. Microcirculation.

[153] Ko K., Suzuki T., Ishikawa R., Hattori N., Ito R., Umehara K., Furihata T., Dohmae N., Linhardt R.J., Igarashi K. (2020). Ischemic stroke disrupts the endothelial glycocalyx through activation of proHPSE via acrolein exposure. J. Biol. Chem..

[154] Ding Z., Wang X., Khaidakov M., Liu S., Dai Y., Mehta J.L. (2012). Degradation of heparan sulfate proteoglycans enhances oxidized-LDL-mediated autophagy and apoptosis in human endothelial cells. Biochem. Biophys. Res. Commun..

[155] Annecke T., Fischer J., Hartmann H., Tschoep J., Rehm M., Conzen P., Sommerhoff C.P., Becker B.F. (2011). Shedding of the coronary endothelial glycocalyx: Effects of hypoxia/reoxygenation vs. ischaemia/reperfusion. Br. J. Anaesth..

[156] Lee S.-J., Lee C., Kang S., Park I., Kim Y.H., Kim S.K., Hong S.P., Bae H., He Y., Kubota Y. (2018). Angiopoietin-2 exacerbates cardiac hypoxia and inflammation after myocardial infarction. J. Clin. Investig..

[157] Mulivor W., Lipowsky H.H. (2002). Role of glycocalyx in leukocyte-endothelial cell adhesion. Am. J. Physiol. Heart Circ. Physiol..

[158] Dolapoglu A., Avci E. (2024). Relationship between pan-immune- inflammation value and in major cardiovascular and cerebrovascular events in stable coronary artery disease patients undergoing on-pump coronary artery bypass graft surgery. J. Cardiothorac. Surg..

[159] Hassanabad A.F., Schoettler F.I., Kent W.D.T., Adams C.A., Holloway D.D., Ali I.S., Novick R.J., Ahsan M.R., McClure R.S., Shanmugan G. (2023). Cardiac surgery elicits pericardial inflammatory responses that are distinct compared with postcardiopulmonary bypass systemic inflammation. JTCVS Open.

[160] Schädler D., Pausch C., Heise D., Meier-Hellmann A., Brederlau J., Weiler N., Marx G., Putensen C., Spies C., Jörres A. (2017). The effect of a novel extracorporeal cytokine hemoadsorption device on IL-6 elimination in septic patients: A randomized controlled trial. PLoS ONE.

[161] Singh Y., Chhabra S., Lashkari K., Taneja A., Garg A., Chandra A., Chhabra M., Singh G., Jain S. (2020). Hemoadsorption by extracorporeal cytokine adsorption therapy (CytoSorb^®^) in the management of septic shock: A retrospective observational study. Int. J. Artif. Organs.

[162] Ishay S.Y., Abu-Tailakh M., Raichel L., Hershenhoren T.F., Matsa M., Lev-Ran O., Gideon S., Douvdevani A. (2022). A prospective cohort study of dynamic cell-free DNA elevation during cardiac surgery with cardiopulmonary bypass. PLoS ONE.

[163] Loh W., Vermeren S. (2022). Anti-Inflammatory Neutrophil Functions in the Resolution of Inflammation and Tissue Repair. Cells.

[164] McCully M.L., Moser B. (2011). The human cutaneous chemokine system. Fron.t Immunol..

[165] Shafqat A., Khan J.A., Alkachem A.Y., Sabur H., Alkattan K., Yaqinuddin A., Sing G.K. (2023). How Neutrophils Shape the Immune Response: Reassessing Their Multifaceted Role in Health and Disease. Int. J. Mol. Sci..

[166] Oishi Y., Manabe I. (2018). Macrophages in inflammation, repair and regeneration. Int. Immunol..

[167] Lund H., Hunt M.A., Kurtović Z., Sandor K., Kägy P.B., Fereydouni N., Julien A., Goritz C., Vasquez-Liebanas E., Mae M.A. (2024). CD163+ macrophages monitor enhanced permeability at the blood–dorsal root ganglion barrier. J. Exp. Med..

[168] Margraf A., Ludwig N., Zarbock A., Rossaint J. (2020). Systemic Inflammatory Response Syndrome After Surgery: Mechanisms and Protection. Anesth. Analg..

[169] Kefalogianni R., Kamani F., Gaspar M., Aw T.C., Donovan J., Laffan M., Pickering M.C., Arachchillage D.J. (2022). Complement activation during cardiopulmonary bypass and association with clinical outcomes. EJHaem.

[170] Boer C., Meesters M.I., Veerhoek D., Vonk A.B.A. (2018). Anticoagulant and side-effects of protamine in cardiac surgery: A narrative review. Br. J. Anaesth..

[171] Levy J.H., Ghadimi K., Kizhakkedathu J.N., Iba T. (2023). What’s fishy about protamine? Clinical use, adverse reactions, and potential alternatives. J. Thromb. Haemost..

[172] van den Goor J., Nieuwland R., van den Brink A., van Oeveren W., Rutten P., Tijssen J., Eijsman L. (2004). Reduced complement activation during cardiopulmonary bypass does not affect the postoperative acute phase response. Eur. J. Cardiothorac. Surg..

[173] Stahl G.L., Shernan S.K., Smith P.K., Levy J.H. (2012). Complement activation and cardiac surgery: A novel target for improving outcomes. Anesth. Analg..

[174] Wisgrill L., Lamm C., Hell L., Thaler J., Berger A., Weiss R., Weber V., Rinoesl H., Hiesmayr M.J., Spittler A. (2020). Influence of hemoadsorption during cardiopulmonary bypass on blood vesicle count and function. J. Transl. Med..

[175] Wang M., Feng J., Zhou D., Wang J. (2023). Bacterial lipopolysaccharide-induced endothelial activation and dysfunction: A new predictive and therapeutic paradigm for sepsis. Eur. J. Med. Res..

[176] McBride W.T., Armstrong M.A., Crockard A.D., McMurray T.J., Rea J.M. (1995). Cytokine balance and immunosuppressive changes at cardiac surgery: Contrasting response between patients and isolated CPB circuits. Br. J. Anaesth..

[177] Margraf A., Lowell C.A., Zarbock A. (2022). Neutrophils in acute inflammation: Current concepts and translational implications. Blood.

[178] Binda D.D., Baker M.B., Varghese S., Wang J., Badenes R., Bilotta F., Nozari A. (2024). Targeted Temperature Management for Patients with Acute Ischemic Stroke: A Literature Review. J. Clin. Med..

[179] Soares R.O.S., Losada D.M., Jordani M.C., Évora P., Castro-e-Silva O. (2019). Ischemia/Reperfusion Injury Revisited: An Overview of the Latest Pharmacological Strategies. Int. J. Mol. Sci..

[180] Goncharov R.G., Sharapov M.G. (2023). Ischemia–Reperfusion Injury: Molecular Mechanisms of Pathogenesis and Methods of Their Correction. Mol. Biol..

[181] Vlastos D., Zeinah M., Ninkovic-Hall G., Vlachos S., Salem A., Asonitis A., Chavan H., Kalampalikis L., Shammari A.A., Gallesio J.M.A. (2022). The effects of ischaemic conditioning on lung ischemia–reperfusion injury. Respir. Res..

[182] Skrzypczak-Wiercioch A., Sałat K. (2022). Lipopolysaccharide-Induced Model of Neuroinflammation: Mechanisms of Action, Research Application and Future Directions for Its Use. Molecules.

[183] Giacinto O., Satriano U., Nenna A., Spadaccio C., Lusini M., Mastroianni C., Nappi F., Chello M. (2019). Inflammatory Response and Endothelial Dysfunction Following Cardiopulmonary Bypass: Pathophysiology and Pharmacological Targets. Recent Pat. Inflamm. Allergy Drug Discov..

[184] Yu Y., Li C., Zhu S., Jin L., Hu Y., Ling X., Miao C., Guo K. (2023). Diagnosis, pathophysiology and preventive strategies for cardiac surgery-associated acute kidney injury: A narrative review. Eur. J. Med. Res..

[185] Busse L.W., Barker N., Petersen C. (2020). Vasoplegic syndrome following cardiothoracic surgery—Review of pathophysiology and update of treatment options. Crit. Care.

[186] Mensah S.A., Cheng M.J., Homayoni H., Plouffe B.D., Coury A.J., Ebong E.E. (2017). Regeneration of glycocalyx by heparan sulfate and sphingosine 1-phosphate restores inter-endothelial communication. PLoS ONE.

[187] Giantsos-Adams K.M., Koo A.J.-A., Song S., Sakai J., Sankaran J., Shin J.H., Garcia-Cardena G., Dewey C.F. (2013). Heparan Sulfate Regrowth Profiles Under Laminar Shear Flow Following Enzymatic Degradation. Cell Mol. Bioeng..

[188] Targosz-Korecka M., Malek-Zietek K.E., Kloska D., Rajfur Z., Stepien E.Ł., Grochot-Przeczek A., Szymonski M. (2020). Metformin attenuates adhesion between cancer and endothelial cells in chronic hyperglycemia by recovery of the endothelial glycocalyx barrier. Biochim. Biophys. Acta (BBA)-Gen. Subj..

[189] Long R., Vink H. (2016). (Microvascular Health Solutions LLC). Synergistic Glycocalyx Treatment Compositions and Methods. U.S. Patent.

[190] Doherty M., Buggy D.J. (2012). Intraoperative fluids: How much is too much?. Br. J. Anaesth..

[191] Aldecoa C., Llau J.V., Nuvials X., Artigas A. (2020). Role of albumin in the preservation of endothelial glycocalyx integrity and the microcirculation: A review. Ann. Intensive Care.

[192] Zuurbier C.J., Demirci C., Koeman A., Vink H., Ince C. (2005). Short-term hyperglycemia increases endothelial glycocalyx permeability and acutely decreases lineal density of capillaries with flowing red blood cells. J. Appl. Physiol..

[193] Barelli S., Alberio L. (2018). The role of plasma transfusion in massive bleeding: Protecting the endothelial glycocalyx?. Front. Med..

[194] Caraceni P., Tufoni M., Bonavita M.E. (2013). Clinical use of albumin. Blood Transfus. Trasfus. Sangue..

[195] Lin M.C., Lin C.F., Li C.F., Sun D.P., Wang L.Y., Hsing C.H. (2015). Anesthetic propofol overdose causes vascular hyperpermeability by reducing endothelial glycocalyx and ATP production. Int. J. Mol. Sci..

[196] Orriach J.G., Ortega M.G., Fernandez A.R., Aliaga M.R., Cortes M.M., Villanueva D.A., Vela A.F., Torres J.A., Fernandez C.S., Gonzalez E.M. (2017). Cardioprotective efficacy of sevoflurane vs. propofol during induction and/or maintenance in patients undergoing coronary artery revascularization surgery without pump: A randomized trial. Int. J. Cardiol..

[197] O’Hora T.R., Markos F., Wiernsperger N.F., Noble M.I. (2012). Metformin causes nitric oxide-mediated dilatation in a shorter time than insulin in the iliac artery of the anesthetized pig. J. Cardiovasc. Pharmacol..

[198] Cooper S., Teoh H., Campeau M.A., Verma S., Leask R.L. (2019). Empagliflozin restores the integrity of the endothelial glycocalyx in vitro. Mol. Cell Biochem..

